# Association of Perioperative Regional Analgesia with Postoperative Patient-Reported Pain Outcomes and Opioid Requirements: Comparing 22 Different Surgical Groups in 23,911 Patients from the QUIPS Registry

**DOI:** 10.3390/jcm10102194

**Published:** 2021-05-19

**Authors:** Marcus Komann, Alexander Avian, Johannes Dreiling, Hans Gerbershagen, Thomas Volk, Claudia Weinmann, Winfried Meißner

**Affiliations:** 1 Department of Anesthesiology and Intensive Care Medicine, Jena University Hospital, 07747 Jena, Germany; Johannes.dreiling@med.uni-jena.de (J.D.); Claudia.weinmann@med.uni-jena.de (C.W.); winfried.meissner@med.uni-jena.de (W.M.); 2 Institute for Medical Informatics, Statistics and Documentation, Medical University of Graz, 8036 Graz, Austria; alexander.avian@medunigraz.at; 3 Klinik für Anästhesiologie, Operative Intensivmedizin, Notfallmedizin und Schmerztherapie, Marienhospital Gelsenkirchen, 45886 Gelsenkirchen, Germany; hans.gerbershagen@gmail.com; 4 Klinik für Anästhesiologie, Intensivmedizin und Schmerztherapie, Universitätsklinikum des Saarlandes, 66421 Homburg, Germany; Thomas.Volk@uks.eu

**Keywords:** regional anesthesia, RA, acute pain, postoperative pain, pain management, functional impairment, opioid consumption

## Abstract

(1) Background: In many surgical procedures, regional analgesia (RA) techniques are associated with improved postoperative analgesia compared to systemic pain treatment. As continuous RA requires time and experienced staff, it would be helpful to identify settings in which continuous RA has the largest benefit. (2) Methods: On the basis of 23,911 data sets from 179 German and Austrian hospitals, we analyzed the association of perioperative RA with patient-reported pain intensity, functional impairment of movement, nausea and opioid use for different surgeries. Regression analyses adjusted for age, sex and preoperative pain were performed for each surgery and the following groups: patients receiving continuous RA (surgery and ward; RA++), RA for surgery only (RA+−) and patients receiving no RA (RA−−). (3) Results: Lower pain scores in the RA++ compared to the RA−− group were observed in 13 out of 22 surgeries. There was no surgery where pain scores for RA++ were higher than for RA−−. If maximal pain, function and side effects were combined, the largest benefit of continuous RA (RA++) was observed in laparoscopic colon and sigmoid surgery, ankle joint arthrodesis, revision (but not primary) surgery of hip replacement, open nephrectomy and shoulder surgery. The benefit of RA+− was lower than that of RA++. (4) Discussion: The additional benefit of RA for the mentioned surgeries is larger than in many other surgeries in clinical routine. The decision to use RA in a given surgery should be based on the expected pain intensity without RA and its additional benefits.

## 1. Introduction

Regional analgesia (RA) techniques are associated with improved postoperative analgesia compared to systemic pain treatment in a large number of surgical procedures [1,2,3]: “The universal efficacy of epidural analgesia has been well demonstrated. Regardless of analgesic agent used, location of catheter, type of surgery and type or time of pain assessment, epidural analgesia often provides better pain relief than parenteral opioid administration”. Moreover, some studies indicate an association between use of epidural analgesia and improved outcome and reduced risk of chronic postoperative pain in specific patient populations. Consequently, international guidelines recommend epidural and peripheral blocks for postoperative pain management for many orthopedic and large abdominal and thoracic operations [4].

However, compared to most forms of systemic pain management, continuous RA requires experienced clinicians for catheter placement, intensive patient monitoring, increased staff resources and medical devices. Furthermore, it places patients at specific risks like infection, hematoma, and toxicity of local anesthetics and opioids.

As resources to provide RA are limited in most hospitals, they should be allocated to those settings and patients with the highest possible benefit. Therefore, it would be valuable to compare the probable added benefit of RA between different types of surgeries. However, this is hardly possible on the basis of single RCTs as they are performed in different settings, differ in interventions and control groups, and often use heterogeneous methodology of outcome assessment, thus making it difficult to compare their results.

On the basis of a large German postoperative patient registry, we compared differences in patient-reported pain intensity, function, nausea and opioid use between patients with and without regional analgesic treatment in different groups of surgeries.

## 2. Materials and Methods

### 2.1. Data Registry

Our analysis is based on data from the ‘Quality Improvement in Postoperative Pain Treatment’ project (QUIPS), the German part of the international PAIN OUT database. The core of this project is a large database that allows comparison of patient-reported outcomes (PROs) of pain parameters in German and Austrian hospitals [5]. Hospitals participate on a voluntary basis to improve the outcome of their postoperative pain management. They pay an administration fee and receive web-based, anonymized feedback of their results. The project is run by the German and Austrian Societies of Anesthesiologists and Surgeons. It is approved by Jena University Hospital’s ethics committee (approval number 2722-12/09). PROs are obtained by use of the validated 15-item QUIPS questionnaire [6,7].

The questionnaire comprises maximum, least and movement-evoked pain since surgery as well as pre-existing chronic pain intensity (for three months) using a numeric rating scale (NRS) of 0–10 (0 = no pain, 10 = worst pain imaginable). In addition, pain-related interference with respiration, sleep, movement, mood; nausea, vomiting and tiredness using a dichotomous scale (yes/no); and demographic and clinical data (age, gender, type of surgery, anesthesia, and pain treatment, including use of systemic or regional analgesia on wards) are assessed. Type of surgery was coded according to the German OPS system.

Data were collected by site staff after they had passed half-day training. This training included the standard operating procedures for data collection, randomization of patients, and standardized obtaining and recording of data. Based on a written information sheet, patients were informed that participation was voluntary and could be withdrawn any time without negative consequences. Patients were asked to provide informed consent as determined by the local institutional review board or ethics committee.

Patients had to meet the following inclusion criteria: age 18 years or older, able to communicate, not cognitively impaired, not sedated or sleeping and willing to participate.

Patients completed the questionnaire on their own. They were only assisted if they requested help or if they were physically unable to complete the survey, e.g., because they had a broken wrist. Data were inputted by data collection personnel via an SSL-secured, Internet-accessible webform. It was stored on a server in the demilitarized zone of Jena University Hospital. Patient information is pseudonymized.

### 2.2. Selection of Data Sets and Grouping of Surgeries

Surgeries were grouped similar to the method described by Gerbershagen et al. in 2013 [8]. Briefly described, all operation and procedure codes (OPS codes) indicating minor variations of a surgery were assigned to one top-level surgery category (e.g., open hysterectomy with resection of the uterus alone as well as with single side or bilateral salpingoovarektomy was grouped as “open hysterectomy”). Open and laparoscopic access variants were regarded as separate surgery categories.

For primary analysis, data sets were distinguished according to the QUIPS data items “regional anesthesia for surgery? (y/n)” and “regional analgesia on ward? (y/n)” into patients receiving RA for surgery and on the ward (RA++), patients receiving RA for surgery but not on the ward (RA+−) and patients receiving RA neither for surgery nor on the ward (RA−−). None of our patients received RA on the ward only.

Surgery categories were considered for analysis if they comprised a minimum of 20 cases in the RA−− group and at least 20 cases either from the RA+− or from the RA++ group. The minimum proportion of cases with RA within an analysis had to be at least 5% of all surgeries in a group.

### 2.3. Data Analysis

We analyzed the following PROs as main outcome parameters: Worst pain (representing pain intensity), number of patients reporting pain-associated impairment of mobilization (representing function) and number of patients reporting nausea (representing side effects). Second, opioid use was analyzed. All patients with at least one dose of opioids given on ward were defined as opioid users. Non-opioid use was defined if the box “no opioid given” was ticked.

To analyze the potential association of RA with pain, function, side effects and on opioid use, regression analyses adjusted for age, sex and preoperative pain [9] were performed separately for each of the different surgical categories and for the following groups: RA++ vs. RA−−, RA+− vs. RA−−. Due to the ongoing debate of pain intensity numeric rating scale (NRS) being ordinal or ratio scale [10], the influence on worst pain was analyzed using linear regression as well as ordinal regression analyses. Linear regression analysis was adjusted for age and sex. RA was the last variable entered in the model and therefore R^2^ changes to the final model were used to calculate Cohen’s d. Furthermore, regression coefficients (β) for RA and corresponding 95% confidence intervals (95%CI) were reported. To analyze the impact on pain-associated impairment of mobilization, nausea and opioid use (yes/no), logistic regression analyses (age and sex adjusted) were performed. Odds ratios and 95%CI were reported. Since surgery categories differ in sample size, all effects d > 0.2 regardless of p-value were also reported. According to the definition of Cohen an effect sizes of d = 0.2 is small, d = 0.5 medium and d = 0.8 large [11]. Therefore, we considered d ≤ 0.35 a small effect size, d > 0.35 to d ≤ 0.65 a medium effect and d > 0.65 strong effect. To get an overall impression of all three primary outcomes, we combined the effects of RA on pain, function and side effects. Odds ratios of function and side effects were converted into effect sizes. Together with effect sizes of pain intensity, results were averaged and ranked.

In some surgeries, different types of RA were used. Therefore, we performed a sub-analysis comparing peripheral and neuraxial blockade techniques in hip and knee replacement.

Data were not imputed. Only data sets without missing values in the primary outcomes were used. A *p*-value less than 5% was considered significant. For data analysis, IBM SPSS Statistics 22 (IBM Corporation, Armonk, NY, USA; 2013) and SAS 9.4 (SAS Institute Inc., Cary, NC, USA; 2012) were used.

Selection of data, surgical grouping, choice of outcome parameters and the statistical approach, including effect sizes, were defined a priori. The decision to differentiate between neuraxial and peripheral blockade techniques in selected surgeries was done during the analysis.

## 3. Results

A total of 189,774 data sets were exported from the QUIPS registry. 17,214 had to be excluded because of missing OPS, 20,889 because of missing primary outcome variables, and 27 surgical categories could be identified. One out of these 27 surgery categories did not fulfil the inclusion criteria because of the low number of patients (*n* < 20) in the RA−− group (gastrectomy), and another one because of the low number of patients in both the RA++ and RA+− group (open left hemicolectomy). Three surgery categories were excluded because of the low percentage of patients (<5%) in the RA+− and RA++ group (vaginal hysterectomy, lap. hysterectomy and open cholecystectomy: <5% RA), leaving 22 surgery categories for comparison RA−− with RA++. For the comparison of RA−− and RA+−, eight surgery categories were excluded because of the low number of patients in the RA+− group (open hysterectomy, liver resection, open right hemicolectomy, lap. partial colon resection, open sigmoidectomy, open rectum resection and open nephrectomy).

Number of patients varied between 31 (arthrodesis ankle joint, RA+− group) and 4099 (hip joint replacement, RA−− group). In total, 23,911 data sets from 179 different hospitals with completed PROs were included in the analysis. Overall, 9133 patients received RA for surgery and on the ward (RA++), 5259 received RA for surgery only (RA+−) and 9519 did not receive RA at all (RA−−). In 129 patients, documentation on RA during surgery was missing. Patients’ characteristics and surgical outcome parameters are shown in Table 1 and Table 2.

### 3.1. Continuous RA vs. No RA (RA++ vs. RA−−)

**Figure 1:**
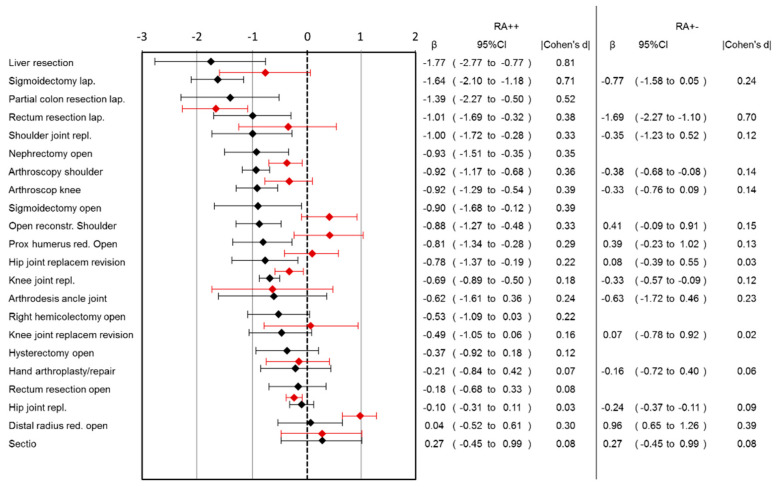
Association of RA with pain intensity in different surgical categories. Patients with continuous (RA++; black lines) or single-shot RA (RA+−; red lines) are compared to patients without RA (RA−−). Pain was measured on an 11-step numeric rating scale (NRS). Effect sizes (β) and corresponding 95% confidence intervals and absolute effect sizes (|Cohen’s d|) for both analyses are shown.

Lower pain scores in the RA++ group were observed in 13 out of 22 surgery categories (Figure 1). The largest benefit was observed for liver resection (pain reduction: 1.77, 95%CI: 0.77–2.77), followed by laparoscopic sigmoidectomy, laparoscopic rectum resection and shoulder joint replacement. There was no surgical category where pain scores for RA++ were higher than for RA−−. Ordinal regression analysis showed comparable results to linear regression analysis (Supplemental Table S1).

**Figure 2:**
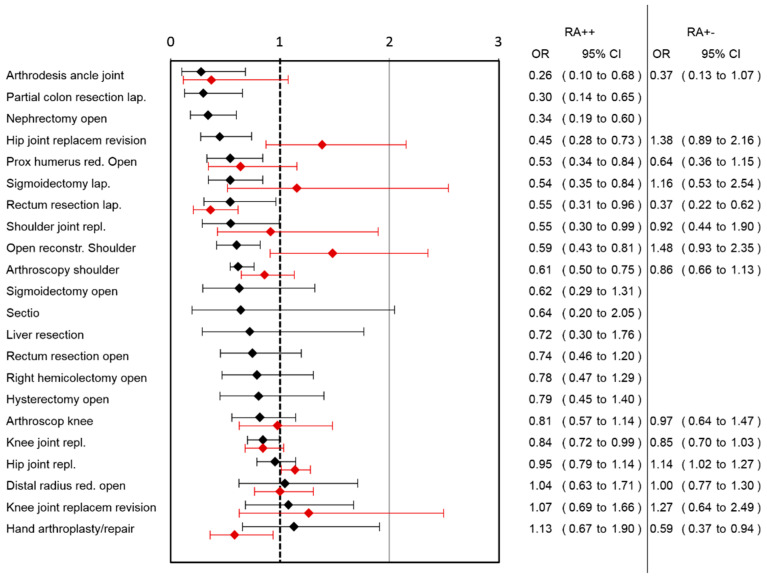
Association of RA with pain-associated impairment of mobilization in different surgical categories. Patients with continuous (RA++; black lines) or single-shot RA (RA+−; red lines) are compared to patients without RA (RA−−). OR and corresponding 95% confidence intervals for both analyses are shown.

A lower risk for pain-associated interference with mobilization was observed in the RA++ group after arthrodesis of the ankle joint, laparoscopic partial colon resection, open nephrectomy, hip joint replacement revision, open reduction of the proximal humerus, laparoscopic sigmoidectomy, laparoscopic rectum resection, shoulder joint replacement, open reconstruction of the shoulder, arthroscopic shoulder surgery and knee joint replacement (Figure 2). A higher risk for impaired mobilization in RA++ was not observed in any of the surgical categories.

**Figure 3:**
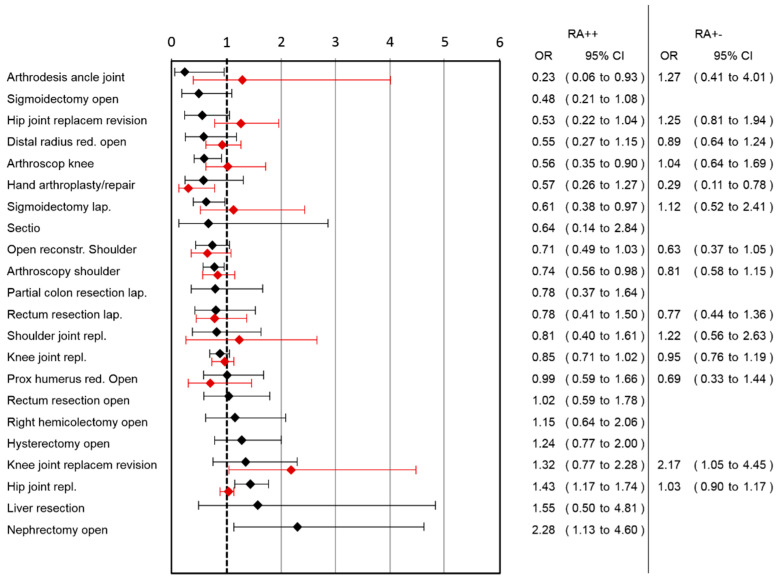
Association of RA with nausea in different surgical categories. Patients with continuous (RA++; black lines) or single-shot RA (RA+−; red lines) are compared to patients without RA (RA−−). OR and corresponding 95% confidence intervals for both analyses are shown.

A lower risk for nausea was observed in the RA++ group after arthrodesis of the ankle joint, arthroscopy of the knee, laparoscopic sigmoidectomy and arthroscopic shoulder surgery, while a higher risk in hip joint replacement and open nephrectomy was observed (Figure 3).

**Figure 4:**
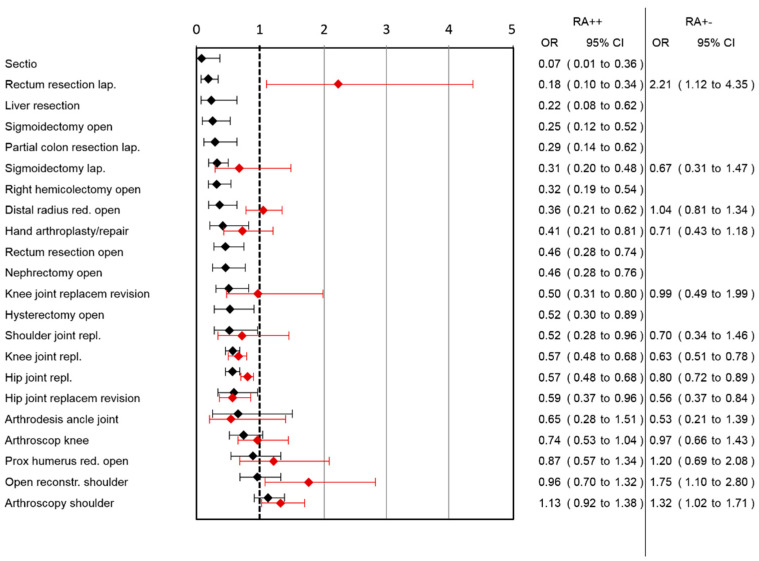
Association of RA with opioid use in different surgical categories. Patients with continuous (RA++; black lines) or single-shot RA (RA+−; red lines) are compared to patients without RA (RA−−). OR and corresponding 95% confidence intervals for both analyses are shown.

A lower risk for opioid use was observed in 16 out of 21 surgery categories (Figure 4) (two surgery category section could not be analyzed because of the low number of patients in the RA++ group). The highest effect was observed in the surgery category caesarean section, followed by laparoscopic rectum resection, followed by liver resection, open sigmoidectomy and laparoscopic partial colon resection.

If worst pain, pain-associated interference with mobilization and nausea were combined, the largest advantages of RA++ could be observed in the categories of arthrodesis of the ankle joint, laparoscopic partial colon resection, laparoscopic sigmoidectomy and open sigmoidectomy (Supplemental Table S2).

### 3.2. Intraoperative RA vs. No RA (RA+− vs. RA−−)

Comparing RA+− group with RA−−, the largest benefit of RA in pain reduction was observed in lap. rectum resection (pain reduction: 1.69, 95%CI: 1.1–2.27) followed by arthroscopic shoulder surgery, knee joint replacement and hip joint replacement (Figure 1). In the RA+− group, higher pain scores were observed in the open reduction of the distal radius surgery category. Ordinal regression analysis showed comparable results to linear regression analysis (Supplemental Table S3). A lower risk for pain-associated interference with mobilization was observed in the RA+− group after lap. rectum resection and hand arthroplasty/repair, while a higher risk after hip joint replacement was observed (Figure 2). In the RA+− group, a lower risk for nausea was observed after hand arthroplasty/repair and a higher risk after knee joint replacement revision (Figure 3). In the RA+− group, risk for opioid use was reduced after hip joint replacement revision, knee joint replacement and hip joint replacement (Figure 4). An increased risk was observed after arthroscopic shoulder surgery, open reconstruction of the shoulder and lap. rectum resection. A combined medium effect of RA++ on worst pain, pain-associated interference with mobilization and nausea could be observed for lap. rectum resection (Supplemental Table S4).

### 3.3. Peripheral vs. Neuroaxial Blockade Techniques in Hip and Knee Replacement on the Ward

In hip joint replacement, neuraxial blockade was associated with a higher risk of nausea compared to the RA−− group (OR: 1.70; 95%CI: 1.29–2.24).

In knee joint replacement, peripheral blockade was superior to neuraxial blocks and no RA was documented in the patient-reported outcomes of pain intensity and pain-associated impairment of mobilization. No differences were found for nausea. Patients with neuraxial techniques had a higher risk of impaired mobilization than patients without RA (see Supplementary Table S5).

Opioid use was lower in all types of RA and in both surgical groups compared to the RA−− group (see Supplementary Table S6).

## 4. Discussion

We compared the impact of RA on pain intensity, interference with mobilization, nausea and opioid requirements between different types of surgery in clinical routine. We used a highly standardized assessment of these outcomes in almost 24,000 patients. Overall, RA was superior to no RA for the outcomes of pain, function and opioid use in many surgical categories. However, the extent of benefit differed considerably between analyzed surgeries.

If impact on pain, function and side effects were combined, the largest benefit of continuous RA (RA++) was observed in laparoscopic colon and sigmoid surgery, ankle joint arthrodesis, revision surgery of hip replacement, open nephrectomy, and shoulder surgery (including arthroscopy).

One major finding of this study suggests that the additional benefit achievable with RA after laparoscopic colon and sigmoid surgery exceeds the effects of RA in many other types of surgery (including open surgery) which contrasts common assumption. Often, laparoscopic surgery is considered to be less painful than open techniques, and the benefit of RA techniques is questioned in some RCTs [12,13]. However, a recent analysis of an international database using a matched-pair design showed also a large benefit of epidural analgesia for both laparoscopic and open visceral surgery [14]. We hypothesize that our findings mirror the underestimation of pain after “non-invasive” surgical techniques, resulting in inadequate systemic pain management and care of these patients. Therefore, our results do not necessarily call for use of more RA in these patients, but for more awareness and standardized pharmacological and non-pharmacological treatment protocols after these surgeries.

In primary hip replacement, RA did not improve postoperative pain management in contrast to hip replacement revision, where patient-reported pain, impairment of mobilization and nausea were significantly improved in patients with continuous RA compared to those patients without RA. Recent guidelines do not differentiate between primary and revision hip surgery [4].

Ankle joint arthrodesis was the most painful surgery in our analysis, and RA improved the combined PROs of pain (although still not to a satisfactory level), pain-related interference with mobilization and nausea more than in any other surgical category. These findings support recommendations to increase RA use in this type of surgery [15,16].

Shoulder surgery (both open reconstruction and arthroscopy) represented another surgical group with a large additional benefit when using RA postoperatively. However, regional anesthesia is still largely underused in shoulder arthroscopy [17].

Open nephrectomy is the most painful surgery in urology [8]. Neither recently published guidelines nor summaries of evidence for acute pain management [4,18] mention this operation. Our data show a large improvement in pain intensity and function (but not in nausea) in those patients receiving RA compared to those who do not.

In almost all types of surgery, opioid use was lower in the RA group. This finding confirms that pain reduction was not caused by increased systemic analgesics and underlines the analgesic potency of RA. Although opioid reduction is not an end in itself, reduction in nausea in some surgical categories suggests a clinically relevant impact.

In many surgical groups, using RA only during surgery (RA+−) improved PROs to some extent in comparison to no RA (RA−−). But the results improved even more when RA was continued after surgery (RA++). Therefore, patients after single-shot RA should be carefully monitored and timely treated when the effects of RA are fading out (which often will happen at night).

Regional analgesia demands higher personnel and technical effort than systemic analgesia [19,20,21], and many hospitals have limited capacities for acute pain management. Therefore, it would be helpful to allocate resources to provide regional analgesia to those patients who will benefit most. However, the overall clinical relevance of a specific form of pain management in a given patient depends on the absolute level of pain (e.g., a reduction of pain intensity from 6 to 4.5 NRS might be more relevant than from 2 to 0.5 NRS), on its impact on functional interference and side effects, and on the balance between risks and benefit. Furthermore, it is influenced by many individual and hospital-specific factors (e.g., psychological variables, patients’ preferences, pre-existing pain, staff experience with specific treatments etc.). Therefore, our results should not be translated word-for-word into daily routine, but we recommend to measure and to compare outcomes in the individual clinical setting. If deficits are identified, our findings might help to decide where (resource-demanding) RA techniques should be applied.

## 5. Limitations

The participation in the QUIPS registry is voluntary. A selection bias is possible but unlikely due to size and heterogeneity of the registry (different hospital sizes and types, surgery distributions). This study observed postoperative pain management in a large number of patients and institutions in their clinical routine. Treatment techniques were not standardized. Therefore, reported differences may be influenced by variation of quality of care between hospitals, and treatment changes over time. However, this limitation concerns all surgical categories, and both regional and systemic analgesia. Thus, its impact on reported differences might be moderate. Further, the high number of participating hospitals might decrease this bias.

Data collection in the QUIPS registry is standardized but data quality might still be poorer than in prospective controlled trials. The lack of randomization does not allow to draw conclusions on causality. Although we adjusted for a couple of potential factors that might bias our findings, the selection of these factors in our analysis might have missed out on important information, e.g., patient history (including medication), comorbidities, psychosocial aspects, hospital structures, behavior/communication of staff, etc.

The QUIPS registry only measures PROs on the first day after surgery. Surgeries performed later in the day may have single-shot residuals lasting until the next day if long-acting local anesthetics are used. In our analysis, this could make differences between RA+− and RA++ appear smaller than they really are. Further, as catheter-based RA techniques typically are used for at least 2–3 postoperative days, we unfortunately cannot extrapolate the associations beyond the first day.

We decided to include all surgical categories with at least 20 patients per group (with RA and without RA), and the number of RA cases had to be 5% or more per surgical group. This approach may appear arbitrary and resulted in large differences in case numbers, as well as in differences in distribution, confidence intervals and statistical power. But it allows comparing RA effectiveness between many different operations and also in surgeries where RA is rarely performed.

We chose to present all surgeries ranked by the magnitude of their impact on pain, function and nausea. This allows the reader to get an impression on variation of data and to judge on their clinical relevance.

## Figures and Tables

**Table 1:** Patients’ characteristics.

	**RA-Groups**		
	RA−−	RA+−	RA++
	*n* = 9519	*n* = 5259	*n* = 9133
	*n* (%)	*n* (%)	*n* (%)
	or	or	or
	median (IQR)	median (IQR)	median (IQR)
**Age groups**			
18–20	71 (0.7)	41 (0.8)	43 (0.5)
21–30	298 (3.1)	224 (4.3)	176 (1.9)
31–40	369 (3.9)	267 (5.1)	231 (2.5)
41–50	1051 (11.0)	420 (8.0)	762 (8.3)
51–60	1870 (19.6)	964 (18.3)	1838 (20.1)
61–70	2456 (25.8)	1401 (26.6)	2618 (28.7)
71–80	2718 (28.6)	1555 (29.6)	2899 (31.7)
81–90	653 (6.9)	374 (7.1)	551 (6.0)
91–100	32 (0.3)	13 (0.2)	15 (0.2)
>100	1 (0.0)	-	-
**Sex**			
female	5531 (58.1)	3121 (59.3)	5153 (56.4)
male	3988 (41.9)	2138 (40.7)	3980 (43.6)
**Pre-existing chronic pain**			
no	3758 (39.5)	1764 (33.5)	2748 (30.1)
yes	5761 (60.5)	3495 (66.5)	6385 (69.9)
**Surgical categories**			
Hysterectomy open	378 (4.0)	-	69 (0.8)
Sectio	37 (0.4)	258 (4.9)	25 (0.3)
Liver resection	41 (0.4)	-	39 (0.4)
Right hemicolectomy open	127 (1.3)	-	138 (1.5)
Partial colon resection lap.	81 (0.9)	-	56 (0.6)
Sigmoidectomy open	75 (0.8)	-	56 (0.6)
Sigmoidectomy lap.	199 (2.1)	35 (0.7)	168 (1.8)
Rectum resection open	112 (1.2)	-	184 (2.0)
Rectum resection lap.	142 (1.5)	113 (2.1)	85 (0.9)
Nephrectomy open	92 (1.0)	-	228 (2.5)
Shoulder joint repl.	85 (0.9)	87 (1.7)	185 (2.0)
Open reconstr. Shoulder	295 (3.1)	136 (2.6)	403 (4.4)
Arthroscopic shoulder	855 (9.0)	353 (6.7)	777 (8.5)
Open red prox. humerus	296 (3.1)	66 (1.3)	131 (1.4)
Open red distal radius	588 (6.2)	420 (8.0)	76 (0.8)
Hand arthroplasty/repar	218 (2.3)	124 (2.4)	94 (1.0)
Hip joint replacem	4099 (43.1)	2196 (41.8)	593 (6.5)
Hip joint replacem revision	460 (4.8)	141 (2.7)	83 0.9)
Knee joint replacem	820 (8.6)	1056 (20.1)	5056 (55.4)
Knee joint replacem revision	127 (1.3)	69 (1.3)	353 (3.9)
Arthroscopic knee	322 (3.4)	174 (3.3)	287 (3.1)
Arthrodesis ancle joint	70 (0.7)	31 (0.6)	47 (0.5)
**Duration of surgery (minutes)**	76 (56–103)	70 (51–93)	85 (65–113)

**Table 2:** Outcome statistics per regional anesthesia group.

	**RA-Groups**		
	RA−−	RA+−	RA++
	*n* = 9519	*n* = 5259	*n* = 9133
	*n* (%)	*n* (%)	*n* (%)
	or	or	or
	median (IQR)	median (IQR)	median (IQR)
**Worst pain (NRS: 0–10)**	5 (3–7)	5 (3–7)	5 (3–7)
**Pain-associated impairment of mobilization**			
no	3086 (32.4)	1713 (32.6)	3435 (37.6)
yes	6433 (67.6)	3546 (67.4)	5698 (62.4)
**Nausea**			
no	7414 (77.9)	4203 (79.9)	7356 (80.5)
yes	2105 (22.1)	1056 (20.1)	1777 (19.5)
**Opioid use**			
no	3586 (37.7)	2133 (40.6)	3913 (42.8)
yes	5483 (57.6)	2958 (56.2)	4891 (53.6)
missing	450 (4.7)	168 (3.2)	329 (3.6)

## Data Availability

Data availability is restricted to active QUIPS participants according to the QUIPS participation contract and publication strategy.

## Supplementary Materials

The following are available online at https://www.mdpi.com/article/10.3390/jcm10102194/s1, Supplemental Table S1: Combined effect of RA++ vs. RA−− on worst pain, pain-associated impairment of mobilization and nausea in different surgical categories. The effect of RA++ vs. RA−− on opioid use is also shown. Supplemental Table S2: Effect of RA on pain intensity in different surgical categories according to ordinal regression results. Patients with continuous RA (RA++) are compared to patients without RA (RA−−). Supplemental Table S3: Combined effect of RA+- vs. RA-- on worst pain, pain-associated impairment of mobilization and nausea in different surgical categories. The effect of RA+− vs. RA−− on opioid use is also shown. Supplemental Table S4: Effect of RA on pain intensity in different surgical categories according to ordinal regression results. Patients with single-shot RA (RA+−) are compared to patients without RA (RA−−). Supplemental Table S5: Worst pain (mean ± SD), pain associated impairment of mobilization (%) and nausea (%) for all RA- groups and surgeries. Supplemental Table S6: Effect of peripheral vs. neuroaxial blockade techniques in hip and knee replacement on worst pain, nausea and pain-associated impairment of mobilization. Supplemental Table S7: Effect of peripheral vs. neuroaxial blockade techniques in hip and knee replacement on opioid use.
