# Supplementary material for: Association of Perioperative Regional Analgesia with Postoperative Patient-Reported Pain Outcomes and Opioid Requirements: Comparing 22 Different Surgical Groups in 23,911 Patients from the QUIPS Registry
Source: J Clin Med. 2021 May 19;10(10):2194. doi: 10.3390/jcm10102194 (PMC8160876; doi:10.3390/jcm10102194)
Supplement: Supplementary file 1 [file jcm-10-02194-s001.zip › jcm-1204442-SI.pdf]

Supplemental Tables

Supplemental Table S1: Combined effect of RA++ vs. RA-- on worst pain, pain-associated impairment of mobilization and nausea in different surgical categories. The effect of RA++ vs. RA— on opioid use is also shown.

	worst pain	pain-associated impairment of mobilization		nausea			opioid use	
	Cohen's d	OR	Effect size	OR	Effect size	Combined Effect size	OR	Effect size
Arthrodesis ankle joint	-0.24	0.264	-0.74	0.230	-0.81	-0.59	0.645	-0.24
partial colon resection lap	-0.52	0.297	-0.67	0.775	-0.14	-0.44	0.294	-0.68
Sigmoidectomy lap	-0.71	0.543	-0.34	0.608	-0.27	-0.44	0.312	-0.64
Sigmoidectomy open	-0.39	0.620	-0.26	0.478	-0.41	-0.36	0.245	-0.78
Hip joint replacement revision	-0.22	0.449	-0.44	0.531	-0.35	-0.34	0.593	-0.29
Rectum resection lap	-0.38	0.545	-0.34	0.780	-0.14	-0.28	0.183	-0.94
Arthroscopic knee	-0.39	0.805	-0.12	0.559	-0.32	-0.28	0.740	-0.17
Open reconstr. Shoulder	-0.33	0.588	-0.29	0.713	-0.19	-0.27	0.959	-0.02
Arthroscopic shoulder	-0.36	0.612	-0.27	0.743	-0.16	-0.27	1.126	0.07
shoulder joint replacement	-0.33	0.545	-0.34	0.807	-0.12	-0.26	0.520	-0.36
Liver resection	-0.81	0.720	-0.18	1.553	0.24	-0.25	0.218	-0.84
Open red prox humerus	-0.29	0.534	-0.35	0.992	0.00	-0.21	0.871	-0.08
Nephrectomy open	-0.35	0.335	-0.60	2.281	0.46	-0.17	0.458	-0.43
Sectio	0.08	0.636	-0.25	0.636	-0.25	-0.14	[a]	
Knee joint replacement	-0.18	0.844	-0.09	0.851	-0.09	-0.12	0.572	-0.31
hand arthroplasty/repair	-0.07	1.127	0.07	0.572	-0.31	-0.10	0.414	-0.49
Right hemicolectomy open	-0.22	0.782	-0.14	1.148	0.08	-0.09	0.321	-0.63
Rectum resection open	-0.08	0.738	-0.17	1.021	0.01	-0.08	0.455	-0.44
Hysterectomie open	-0.12	0.794	-0.13	1.241	0.12	-0.04	0.517	-0.36
Open red distal radius	0.30	1.038	0.02	0.552	-0.33	0.00	0.360	-0.56
Knee joint replacemt revision	-0.16	1.068	0.04	1.321	0.15	0.01	0.499	-0.38
Hip joint replacement	-0.03	0.950	-0.03	1.427	0.20	0.05	0.567	-0.31

Reference group: RA--

[a] for the secondary outcome opioid use group size was too small

Supplemental Table S2: Effect of RA on **pain intensity** in different surgical categories according to ordinal regression results. Patients with continuous RA (RA++) are compared to patients without RA (RA--).

	**sign.**	**OR 95%CI**
Arthrodesis ankle joint	0.414	0.752 (0.380 - 1.490)
Sectio	0.542	0.750 (0.298 - 1.890)
Sigmoidectomy open	0.042	0.519 (0.277 - 0.976)
Open reconstr. shoulder	0.000	0.598 (0.456 - 0.784)
Hysterectomy open	0.188	0.736 (0.466 - 1.162)
Sigmoidectomy lap	0.000	0.286 (0.195 - 0.419)
Nephrectomy open	0.002	0.499 (0.323 - 0.771)
Partial colon resection lap.	0.001	0.335 (0.177 - 0.635)
shoulder joint repl.	0.012	0.543 (0.336 - 0.875)
Prox humerus red. open	0.001	0.539 (0.373 - 0.778)
Knee joint replacem	0.000	0.660 (0.580 - 0.752)
Knee joint replacem revision	0.112	0.748 (0.523 - 1.070)
Liver resection	0.001	0.220 (0.092 - 0.522)
Arthroscop knee	0.000	0.501 (0.377 - 0.665)
Arthroscopic shoulder	0.000	0.554 (0.466 - 0.658)
Distal radius red. open	0.779	1.062 (0.698 - 1.616)
Rectum resection lap	0.003	0.467 (0.283 - 0.773)
Hip joint replacement	0.535	0.954 (0.820 - 1.108)
Hip joint replacement revision	0.019	0.609 (0.403 - 0.922)
Right hemicolectomy open	0.052	0.652 (0.424 - 1.003)
Rectum resection open	0.288	0.797 (0.525 - 1.210)
hand arthroplasty/repair	0.612	0.890 (0.568 - 1.396)

Supplemental Table S3: Combined effect of RA+- vs. RA-- on worst pain, pain-associated impairment of mobilization and nausea in different surgical categories. The effect of RA+- vs. RA— on opioid use is also shown.

	**worst pain**	**pain-associated impairment of mobilization**		**nausea**			**opioid use**	
	Cohen's d	OR	Effect size	OR	Effect size	Combined Effect size	OR	Effect size
Rectum resection lap	-0.70	0.371	-0.55	0.772	-0.14	-0.47	2.211	0.44
hand arthroplasty/repair	-0.06	0.588	-0.29	0.289	-0.69	-0.35	0.711	-0.19
Arthrodesis ankle joint	-0.23	0.373	-0.54	1.274	0.13	-0.22	0.534	-0.35
Arthroscopic shoulder	-0.14	0.862	-0.08	0.811	-0.12	-0.11	1.320	0.15
Open red prox humerus	0.13	0.644	-0.24	0.686	-0.21	-0.11	1.197	0.10
Knee joint replacement	-0.12	0.848	-0.09	0.950	-0.03	-0.08	0.634	-0.25
Arthroscop knee	-0.14	0.974	-0.01	1.035	0.02	-0.04	0.972	-0.02
Sigmoidectomy lap	-0.24	1.161	0.08	1.121	0.06	-0.03	0.670	-0.22
shoulder joint repl.	-0.12	0.915	-0.05	1.223	0.11	-0.02	0.703	-0.19
Hip joint replacement	-0.09	1.136	0.07	1.025	0.01	0.00	0.799	-0.12
Open reconstr. Shoulder	0.15	1.476	0.22	0.625	-0.26	0.04	1.751	0.31
Open red distal radius	0.39	1.002	0.00	0.891	-0.06	0.11	1.039	0.02
Hip joint replacement revision	0.03	1.381	0.18	1.251	0.12	0.11	0.559	-0.32
Knee joint replacement revision	0.02	1.267	0.13	2.165	0.43	0.19	0.991	0.00
Reference group: RA--								

Supplemental Table S4: Effect of RA on pain intensity in different surgical categories according to ordinal regression results. Patients with single-shot RA (RA+-) are compared to patients without RA (RA--).

	**sign.**	**OR (95%CI)**
Sectio	0.614	1.170(0.635 - 2.156
Sigmoidectomy lap	0.139	0.614 (0.322 - 1.171)
Rectum resection lap	0.000	0.281 (0.178 - 0.445)
shoulder joint replacement	0.761	0.917 (0.524 - 1.604)
Open reconstr. Shoulder	0.066	1.406 (0.978 - 2.022)
Arthroscopic shoulder	0.020	0.772 (0.621 - 0.960)
Prox humerus red. Open	0.299	1.289 (0.799 - 2.081)
Distal radius red. Open	0.000	2.091 (1.673 - 2.615)
hand arthroplasty/repair	0.432	0.854 (0.577 - 1.265)
Hip joint replacement	0.000	0.845 (0.772 - 0.925)
Hip joint replacement revision	0.720	1.063 (0.762 - 1.482)
Knee joint replacement	0.046	0.849 (0.723 - 0.997)
Knee joint replacement revision	0.733	1.095 (0.650 - 1.844)
Arthroscop knee	0.112	0.768 (0.554 - 1.064)
Arthrodesis ankle joint	0.445	0.739 (0.341 - 1.605)

Supplemental Table S5: Worst pain (mean±SD), pain associated impairment of mobilization (%) and nausea (%) for all RA- groups and surgeries.

	**worst pain**			**pain-associated impairment of mobilization**			**nausea**		
	RA-- mean ± SD	RA+- mean ± SD	RA++ mean ± SD	RA--	RA+-	RA++	RA--	RA+-	RA++
Arthrodesis ankle joint	6.4 ± 2.2	5.8 ± 2.9	5.9 ± 2.9	80%	68%	62%	20%	19%	6%
Sectio	6.1 ± 2.2	6.2 ± 2.1	5.9 ± 2.0	76%	73%	68%	16%	14%	12
Sigmoidectomy open	5.8 ± 2.2		4.9 ± 2.3	64%		54%	35%		23%
Open reconstr. Shoulder	5.8 ± 2.4	6.1 ± 2.5	4.9 ± 2.7	68%	74%	55%	27%	19%	21%
Hysterectomie open	5.7 ± 2.1		5.2 ± 2.4	74%		68%	42%		36%
Sigmoidectomy lap	5.7 ± 2.2	4.9 ± 2.5	4.0 ± 2.3	67%	69%	51%	38%	37%	26%
Nephrectomy open	5.7 ± 2.3		4.7 ± 2.4	79%		56%	14%		23%
partial colon resection lap	5.6 ± 2.5		4.2 ± 2.8	67%		43%	37%		30%
Shoulder joint replacement	5.6 ± 2.3	5.4 ± 3.3	4.8 ± 2.9	71%	72%	61%	20%	22%	18%
Open red prox humerus	5.6 ± 2.3	6.0 ± 2.3	4.9 ± 3.0	75%	67%	63%	20%	15%	21%
Knee joint replacement	5.6 ± 2.3	5.3 ± 2.8	4.9 ± 2.7	69%	66%	65%	22%	21%	19%
Knee joint replacem. revision	5.6 ± 2.8	5.6 ± 3.0	5.1 ± 2.7	69%	74%	70%	17%	29%	20%
Liver resection	5.5 ± 2.4		3.7 ± 2.0	54%		46%	17%		23%
Arthroscop knee	5.5 ± 2.2	5.2 ± 2.4	4.6 ± 2.5	72%	72%	67%	17%	20%	11%
Arthroscopic shoulder	5.4 ± 2.4	5.1 ± 2.5	4.5 ± 2.7	70%	68%	59%	17%	16%	14%
Open red distal radius	5.1 ± 2.4	6.0 ± 2.5	5.1 ± 2.7	63%	62%	63%	19%	18%	12%
Rectum resection lap	5.0 ± 2.4	3.3 ± 2.3	3.8 ± 2.5	63%	39%	46%	34%	28%	25%
Hip joint replacement	4.9 ± 2.4	4.6 ± 2.5	4.8 ± 2.6	68%	70%	67%	21%	21%	28%
Hip joint replacem. revision	4.9 ± 2.6	5.0 ± 2.3	4.1 ± 2.4	70%	77%	53%	23%	26%	13%
Right hemicolectomy open	4.9 ± 2.3		4.2 ± 2.4	51%		45%	21%		25%
Rectum resection open	4.9 ± 2.0		4.7 ± 2.2	47%		41%	24%		24%
Hand arthroplasty/repair	4.7 ± 2.6	4.4 ± 2. 7	5.0 ± 2.8	49%	35%	59%	14%	4%	11%

Supplemental Table S6: Effect of peripheral vs. neuroaxial blockade techniques in hip and knee replacement on worst pain, nausea and pain-associated impairment of mobilization.

				% (RA++)	worst pain			nausea		pain-associated impairment of mobilization	
	All	RA--	RA++		sign.	Cohen's d	Beta (95% CI)	sign.	OR (95% CI)	sign.	OR (95% CI)
**Hip joint replacem**											
neuroaxial blockade vs. RA-- (Ref)	4361	4099	262	6%	0.206	0.04	-0.20 (-0.50 to 0.11)	<0.001	1.70 (1.29 to 2.24)	0.833	1.03 (0.79 to 1.35)
peripheral blockade vs. RA-- (Ref)	4430	4099	331	7%	0.869	0.00	-0.02 (-0.30 to 0.25)	0.108	1.24 (0.96 to 1.60)	0.364	0.90 (0.71 to 1.14)
peripheral blockade vs. neuroaxial blockade (Ref)	593	262	331	56%	0.748	0.06	0.16 (-0.26 to 0.58)	0.081	0.72 (0.50 to 1.04)	0.378	0.85 (0.60 to 1.21)
**Knee joint replacem**											
neuroaxial blockade vs. RA-- (Ref)	1410	820	590	42%	0.027	0.12	-0.28 (-0.53 to -0.03)	0.407	0.89 (0.69 to 1.17)	0.004	1.43 (1.12 to 1.82)
peripheral blockade vs. RA-- (Ref)	5056	784	4272	84%	<0.001	0.20	-0.75 (-0.94 to -0.55)	0.078	0.85 (0.70 to 1.02)	0.005	0.79 (0.68 to 0.93)
peripheral blockade vs. neuroaxial blockade (Ref)	5056	590	4466	88%	<0.001	0.12	-0.48 (-0.71 to -0.26)	0.456	0.92 (0.74 to 1.15)	<0.001	0.54 (0.44 to 0.66)

Reference categories are marked with (Ref).

Supplemental Table S7: Effect of peripheral vs. neuroaxial blockade techniques in hip and knee replacement on opioid use.

	RA+- n	RA+- %	sign.	OR (95% CI)
**Hip joint replacem**				
neuroaxial blockade vs. RA-- (Ref)	254	6%	<0.001	0.63 (0.49 - 0.82)
peripheral blockade vs. RA-- (Ref)	321	7%	<0.001	0.52 (0.42 - 0.66)
peripheral blockade vs. neuroaxial blockade (Ref)	321	56%	0.259	0.82 (0.59 - o1.15)
**Knee joint replacem**				
neuroaxial blockade vs. RA-- (Ref)	571	42%	<0.001	0.23 (0.18 - 0.29)
peripheral blockade vs. RA-- (Ref)	4334	85%	<0.001	0.65 (0.54 - 0.77)
peripheral blockade vs. neuroaxial blockade (Ref)	571	12%	<0.001	2.61 (2.19 - 3.12)

Reference categories are marked with (Ref).
